# Fluid Intelligence Predicts Change in Depressive Symptoms in Later Life: The Lothian Birth Cohort 1936

**DOI:** 10.1177/0956797618804501

**Published:** 2018-10-25

**Authors:** Stephen Aichele, Paolo Ghisletta, Janie Corley, Alison Pattie, Adele M. Taylor, John M. Starr, Ian J. Deary

**Affiliations:** 1 Swiss National Centre of Competence in Research LIVES, Universities of Lausanne and Geneva; 2 Faculty of Psychology and Educational Sciences, University of Geneva; 3 Swiss Distance Learning University; 4 Centre for Cognitive Ageing and Cognitive Epidemiology, Department of Psychology, University of Edinburgh; 5 Alzheimer Scotland Dementia Research Centre, University of Edinburgh

**Keywords:** intelligence, depression, longitudinal change, lead-lag, dynamic

## Abstract

We examined reciprocal, time-ordered associations between age-related changes in fluid intelligence and depressive symptoms. Participants were 1,091 community-dwelling older adults from the Lothian Birth Cohort 1936 study who were assessed repeatedly at 3-year intervals between the ages of 70 and 79 years. On average, fluid intelligence and depressive symptoms worsened with age. There was also a dynamic-coupling effect, in which low fluid intelligence at a given age predicted increasing depressive symptoms across the following 3-year interval, whereas the converse did not hold. Model comparisons showed that this coupling parameter significantly improved overall fit and had a correspondingly moderately strong effect size, accounting on average for an accumulated 0.9 standard-deviation increase in depressive symptoms, following lower cognitive performance, across the observed age range. Adjustment for sociodemographic and health-related covariates did not significantly attenuate this association. This implies that monitoring for cognitive decrements in later life may expedite interventions to reduce related increases in depression risk.

Depression and cognitive impairment are adverse mental conditions commonly associated with older age. Both predict reduced quality of life, increased self-neglect and caregiver burden, and heightened mortality risk (Vinkers, Gussekloo, Stek, Westendorp, & van der Mast, 2004). In populations of community-dwelling older adults, prevalence estimates for both depression and cognitive impairment range from 10% to 15% (Cole & Dendukuri, 2003; Petersen et al., 2009). Numerous cross-sectional studies have shown that age-related differences in depressive symptoms and cognitive performance are robustly negatively associated (Perrino, Mason, Brown, Spokane, & Szapocznik, 2008). Longitudinal studies have shown that even a slight reduction in life satisfaction predicts cognitive decline (e.g., Rabbitt, Lunn, Ibrahim, Cobain, & McInnes, 2008) and also that lower baseline cognitive ability predicts poorer prognosis for depression (Vink, Aartsen, & Schoevers, 2008). Yet, to date, there has been very little research on reciprocal associations between changes in cognition and depressive symptoms. This is important because a clearer understanding of temporal precedence in cognition-depression associations may expedite interventions to mitigate age-related worsening in mental health.

Empirical descriptions of these associations are etiologically complex (Bennett & Thomas, 2014). One plausible scenario is that neurodegenerative processes and cerebrovascular disease, as well as related white-matter lesion burden, underlie worsening both in depressive symptoms and in cognitive abilities (Butters et al., 2008). However, some studies have shown that cognition-depression relations persist after statistical adjustment for vascular disease and related risk factors (e.g., Jajodia & Borders, 2011). Genetic traits, such as presence of the epsilon-4 type allele of the apolipoprotein E gene, may also predispose individuals to increased risk for both depression and cognitive impairment (Delano-Wood et al., 2008). Alternatively, greater depressive symptom load may be an independent risk factor for cognitive decline. One possible mechanism would be that depression is associated with neuroendocrine changes similar to those observed in animal models of chronic stress, including hypothalamic-pituitary-adrenal dysregulation, which, if prolonged, can lead to memory deficits (Butters et al., 2008). On the other hand, cognitive decrements could lead to increased difficulty in activities of daily life, such as management of personal finances or social interactions (Baltes & Lang, 1997), which in turn provokes increased depression risk. Some older adults may become increasingly depressed when confronted with their own cognitive decline (Carpenter et al., 2008).

The above scenarios are not mutually exclusive; however, each does imply a different temporal ordering of influence. It may be that cognitive impairment and depression arise concurrently, or elevated depressive symptoms precede cognitive impairment, or cognitive impairment leads to increased risk for depression (Bennett & Thomas, 2014). Longitudinal research of reciprocal associations between depressive symptoms and cognitive performance may therefore shed light on the relative plausibility of these scenarios.

We know of 11 such studies (for a review, see Aichele & Ghisletta, 2018). Six of these used latent growth curve models (LGCMs) to evaluate relations of stability (i.e., baseline levels) and long-term change (i.e., slopes) in depressive symptoms and cognitive performance. But LGCMs do not establish time-ordered effects in the way that lead-lag or dynamic models do (Ghisletta & Lindenberger, 2004). This is an important distinction, particularly in longitudinal observational studies wherein temporal precedence is often implicitly assumed. Also, lead-lag analyses are prone to bias when applied to data at only two time points when variables have different reliabilities (Hamaker, Kuiper, & Grasman, 2015). This leaves three cognition-depression studies that applied lead-lag models to data across three or more time points. Results from these studies showed that lower cognitive performance preceded subsequent increases in depressive symptoms across 1-year intervals (Perrino et al., 2008) and 2-year intervals (Aichele & Ghisletta, 2018; Jajodia & Borders, 2011), whereas the converse did not hold.

More generally, depression risk has most frequently been examined in relation to cognitive decrements indicative of Alzheimer’s disease and vascular dementia (e.g., memory deficits, slowed information processing). However, decrements in abstract reasoning (i.e., fluid intelligence), which are closely related to functional impairment and poor treatment compliance, may better characterize elevated depression risk in nonclinical populations of older adults (Klojcˇnik, Kavcic, & Vukman, 2017). Results from two recent studies support this hypothesis. In a sample of 71 seniors (ages 69–85 years), Klojcˇnik et al. (2017) observed that depression was more strongly associated with decrements in visuospatial reasoning than with decrements in recall memory, verbal fluency, attention, and processing speed. In a longitudinal study of 6,203 middle-age and older adults, Aichele, Rabbitt, and Ghisletta (2017) found that fluid-intelligence decrements better predicted elevated depressive symptoms than did decrements in memory, processing speed, or verbal ability. In an earlier meta-analysis of five UK cohorts of community-based older persons, which included a cohort related to that of the current study, Gale et al. (2011) found that fluid reasoning was significantly negatively associated with depression risk, even after statistical adjustment for verbal intelligence, social class, and health-related variables.

## Scope and Aims of the Current Study

To date, we have been unable to identify a single study of time-ordered, reciprocal associations between fluid intelligence and depressive symptoms. This gap in the literature motivated the current work. In a narrow-age cohort of community-dwelling older adults (*N* = 1,091 at age 70 years; *N* = 550 remaining at age 79 years), measured repeatedly at 3-year intervals between the ages of 70 and 79 years, we used bivariate latent-change-score models (BLCSMs; McArdle, 2001) to examine dynamic associations between age-related changes in fluid intelligence and depressive symptoms. These associations were statistically adjusted for sex, education, smoking status, and concurrent diagnoses of cardiovascular disease, stroke, and diabetes. On the basis of outcomes from previous lead-lag depression-cognition studies (summarized above), we hypothesized that decrements in fluid intelligence at a given age would precede subsequent increases in depressive symptoms, whereas elevated depressive symptoms would not predict subsequent declines in fluid intelligence.

## Method

Data for the analyses came from the Lothian Birth Cohort 1936 (LBC1936) study, which is an ongoing follow-up study of some surviving members of the Scottish Mental Survey 1947 (Scottish Council for Research in Education, 1949). The broader aims and methods of the LBC1936 study have previously been described at length (Deary, Gow, Pattie, & Starr, 2012; Deary et al., 2007; Taylor, Pattie, & Deary, 2018). We therefore briefly summarize participants and measures, whereas we describe the statistical methods in greater detail. All variables (and levels thereof) analyzed for this article’s target research question are reported below. No observations were excluded, nor was a stopping rule imposed on data collection (which remains ongoing, subject to available funding). The large sample size for the study was more than sufficient for estimating the current statistical models.

### Participants

In its first wave of testing, the LBC1936 study included 1,091 participants who had a mean age of 70 years. Most of them had completed a valid test of intelligence (Moray House Test No. 12) as part of the Scottish Mental Survey 1947 at a mean age of 11 years. Most were recruited from the City of Edinburgh and the surrounding area (the Lothians). The study protocol was approved by the Multi-Centre Research Ethics Committee for Scotland (MREC/01/0/56; 07/MRE00/58) and by the Lothian Research Ethics Committee (LREC/2003/2/29). Research was carried out in compliance with the Helsinki Declaration, and all participants gave written, informed consent. The Lothian Health Board made initial contact with potential study candidates (i.e., individuals born in 1936 and who likely completed the Scottish Mental Health Survey in 1947). Of 3,686 individuals identified, 1,703 (46.2%) responded, and 1,091 ultimately became participants following additional screening for eligibility based on year of birth, country of schooling, and being generally healthy enough to attend a hospital research clinic for extensive cognitive and medical evaluation.

### Measures

LBC1936 participants were assessed once between 2004 and 2007 (Wave 1; mean age approximately 70 years; *N* = 1,091) and up to three additional times at approximately 3-year intervals (Waves 2–4; mean ages approximately 73 years, *N* = 866; 76 years, *N* = 697; and 79 years, *N* = 550). Attrition was mainly due to death, chronic incapacity, and permanent withdrawal. Participants were tested individually, at each wave, by a trained psychologist and a research nurse at the Wellcome Trust Clinical Research Facility at the Western General Hospital, Edinburgh. This included cognitive, physical, and other tests (fully described by Taylor et al., 2018). Current analyses used longitudinal data from three measures of fluid intelligence and one measure of depressive symptoms.

#### Fluid intelligence

Fluid intelligence was assessed with three measures of nonverbal ability: Matrix Reasoning and Block Design subtests of the Wechsler Adult Intelligence Scale–Third UK Edition (WAIS-III UK; Wechsler, 1998a) and the Spatial Span (forward and backward) subtest from the Wechsler Memory Scale–Third UK Edition (WMS-III UK; Wechsler, 1998b). The Matrix Reasoning test requires participants to correctly identify missing elements from geometric patterns arranged according to logical rules. In the Block Design test, participants replicate a visuospatial model from component parts within a specified time limit. In the Spatial Span task, participants replicated (recalled) spatial-temporal sequences (2–9 items long) in the same order (forward) or in the reverse order (backward). The Spatial Span score used here was the average of forward and backward performance. Cognitive scores were standardized (within measure, across time) for consistent scaling, using mean and standard deviation at Wave 1 (i.e., score at wave *t* minus mean at Wave 1, divided by standard deviation at Wave 1).

#### Depressive symptoms

Depressive symptoms were measured using the Hospital Anxiety and Depression Scale (HADS; Zigmond & Snaith, 1983), which consists of 14 items (7 related to depressive symptoms, 7 for anxiety). Items are scored in increasing severity from 0 to 3. These scores are summed, giving possible totals of 0 to 21 symptoms for depression and anxiety, respectively. Current analyses used the scores for only the depressive symptoms. A meta-analysis of more than 700 studies using the HADS previously identified a score of 8 as the cutoff for a clinical diagnosis of depression (Bjelland, Dahl, Haug, & Neckelmann, 2002). An advantage of the HADS for the current research objective is that it excludes items related to problems with memory and concentration, which may conflate depressive symptoms with cognitive performance. Previous use of the HADS in community samples of older adults can be found, for example, in Gale et al. (2011).

#### Covariates

We statistically controlled for sex, education level (number of years of formal full-time education), smoking status (never smoked, former smoker, current smoker), social class (based on the Office of Population Censuses and Surveys’ Classification of Occupations, 1980), and the diagnosis at each wave of cardiovascular disease, stroke, and diabetes.

### Statistical analyses

Analyses were based on BLCSMs (McArdle, 2001), which are structural equation models that combine LGCMs (Meredith & Tisak, 1990) and autoregressive cross-lag models (Jöreskog, 1970). LGCMs estimate concurrently variables’ levels and changes (i.e., intercepts and slopes) using information taken from all time points. These static parameters summarize stability and trendlike change across the entire window of observation. In contrast, dynamic cross-lag models estimate time-point-to-time-point couplings between variables, such that variable *X* at time *t* predicts change in variable *Y* between times *t* and *t* + 1, and vice versa. Thus, a dynamic cross-lag study with four measurement occasions would estimate couplings from three sequential change associations. Common cross-lag models assume that variables do not exhibit trendlike change, so data must often be detrended prior to analysis. However, this can bias estimates of association. BLCSMs are designed to overcome this limitation by simultaneously estimating long-term trends and dynamic lead-lag associations. We direct interested readers to Grimm, Ram, and Estabrook (2017), who provide step-by-step guidance for constructing and interpreting BLCSMs. We provide the Mplus code used for the current models in the Supplemental Material available online.

At the measurement level, fluid intelligence was modeled as a latent construct, indicated by performance on the three measures of nonverbal intelligence and estimated under strict factorial invariance (Widaman & Reise, 1997), and applied across measurement occasions, which held statistically (see Table S2 in the Supplemental Material). Depressive symptoms were modeled as an observed variable, scaled in its raw metric. At the structural level of the model, latent change scores (reliable components of changes in fluid intelligence and in depressive symptoms between successive measurement occasions) were specified, from which levels and linear changes (static components), auto-proportional effects (the effect of a variable on its own upcoming change), and coupling effects (the effect of a variable on the upcoming change of the other variable) were estimated. All participants were approximately the same age at testing, so for purposes of interpretability, we refer to the modeled time metric in terms of chronological age rather than measurement occasion.

Model development proceeded in stages. First, we estimated a model (BLCSM0) that included only static parameters (intercept and linear slope) for fluid intelligence and depressive symptoms. This model is statistically equivalent to a bivariate LGCM because it does not estimate any dynamic effects. To test time-dependent coupling effects between fluid intelligence and depressive symptoms, we created four additional models, BLCSM1 through BLCSM4. These latter models included dynamic (auto-proportional and coupling) parameters. We note that the addition of auto-proportional effects respecified static linear changes in cognitive performance and depressive symptoms as nonlinear or exponential changes.

**Fig. 1.**
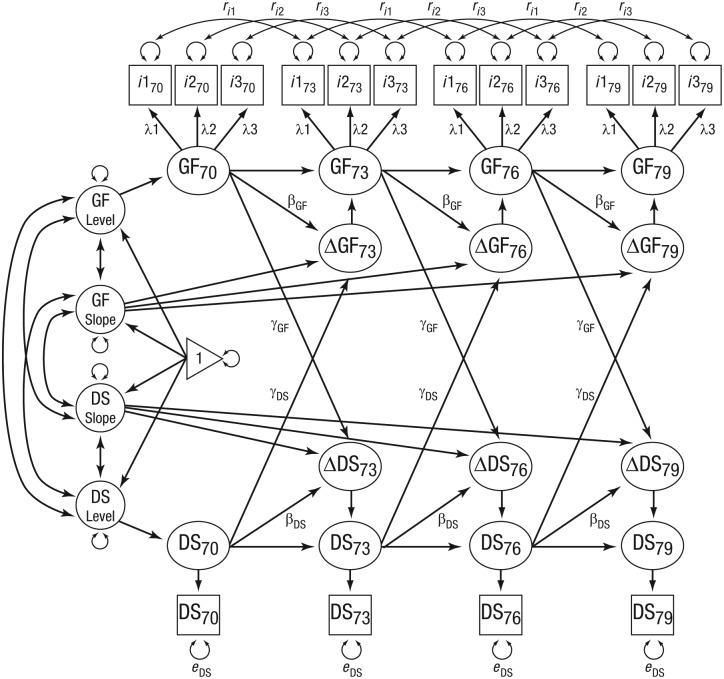
Bivariate latent-change-score model. Fluid intelligence (GF) and depressive symptoms (DS) are modeled across 3-year age intervals (i.e., ages 70, 73, 76, and 79 years—indicated by subscripts). Unlabeled paths with single-headed arrows show regression effects fixed at 1. Unlabeled paths with double-headed arrows show freely estimated variances and covariances. Labels on paths show model-parameter constraints. Time-invariant covariates (e.g., sex, education) and time-varying covariates (e.g., cardiovascular disease) are not shown. Lagged correlations for Gf uniquenesses (*r_i_*s) are shown only for 3-year intervals (Lag 1), but the models also included correlated uniquenesses for 6-year (Lag 2) and 9-year (Lag 3) intervals. Slope = static linear change; *i*1 = Block Design; *i*2 = Matrix Reasoning; *i*3 = Spatial Span observed scores.

For model-comparison purposes, BLCSM1 served as the baseline, or no-coupling, model because it did not include cross-lagged paths between fluid intelligence and depressive symptoms. BLCSM2 was identical to BLCSM1 except for the addition of a unidirectional coupling from fluid intelligence predicting subsequent changes in depressive symptoms. BLCSM3 was identical to BLCSM1 except for the addition of a unidirectional coupling from depressive symptoms predicting subsequent changes in fluid intelligence. Finally, BLCSM4 (see Fig. 1) was the full bidirectional couplings model (i.e., with fluid intelligence → Δdepressive symptoms and depressive symptoms → Δfluid intelligence).

To determine whether couplings were significant, we performed likelihood-ratio tests of change in model fit, as outlined by Grimm et al. (2017), and we examined the couplings’ parameter estimates in the best-fitting model. This was possible because statistically the compared models were nested within the full bidirectional couplings model (BLCSM4). Also, we first ran all models without inclusion of sociodemographic and health variables. We then reran all models adjusting statistically for these covariates, with sociodemographic variables specified as time-invariant predictors of baseline levels and static linear changes for fluid intelligence and depressive symptoms, and with health conditions specified as time-varying predictors of true scores for fluid intelligence and depressive symptoms at each wave.

We chose an alpha criterion of .01 for significance testing of changes in model fit and for parameter estimates. Although studies in the behavioral sciences typically select an alpha criterion of .05, it is not uncommon to find more conservative alpha criteria in population-based lifespan psychology studies, given their relatively large sample sizes (e.g., Aichele & Ghisletta, 2018; Jajodia & Borders, 2011). Selection of each model was guided by its Schwarz weight, that is, its weighted Bayesian information criterion, or w(BIC), rescaled as a relative probability of model preference, as explained by Wagenmakers and Farrell (2004).

We fitted the statistical models using Mplus software (Version 6; Muthén & Muthén, 2012) with full-information maximum-likelihood estimation (FIML). FIML accounts for uncertainty due to missing data, which is especially useful in longitudinal studies in which data are often missing because of attrition, as was the case here (Deary et al., 2012). FIML is tenable when missingness for a given variable does not just depend on that variable itself but can be related to other variables in the model (Schafer & Graham, 2002). This is one reason that we included sociodemographic and health-related covariates in a second series of BLCSM analyses (i.e., as a check against sensitivity to possible sources of missingness). FIML is generally robust to deviations from the assumption that data are normally distributed, especially when using relatively large sample sizes (Hoogland & Boomsma, 1998). Nevertheless, because depressive symptoms were positively skewed, we conducted a follow-up analysis using log-transformed HADS scores as a further check against model fit.

## Results

Raw data are summarized in Tables 1 and 2. On average, fluid intelligence and depressive symptoms worsened between ages 70 and 79 years in both completers (those who remained in the study through Wave 4—about half of the sample) and all comers (everybody). Correlations among observed variables are reported in Table S1 in the Supplemental Material. For all longitudinal measures, within-variable across-wave correlations were, in general, strongest at Lag 1 and fell off slightly at increased lags (i.e., Lags 2 and 3). For cognitive measures, within-variable correlations ranged from .56 (Spatial Span) to .77 (Block Design). Between-variable correlations were strongest between Block Design and Matrix Reasoning (*r*s = .51–.58) and less pronounced between Spatial Span and Block Design (*r*s = .35–.45) and between Spatial Span and Matrix Reasoning (*r*s = .33–.44). Depressive symptoms correlated strongly with themselves across time (*r*s = .61–.73). Within-wave correlations (*r*s) between depressive symptoms and cognitive variables were always negative and became stronger over time, ranging from −.10 (Wave 1) to −.22 (Waves 3 and 4).

**Table 1.** Characteristics of the Sample

Characteristic	All comers (*N* = 1,091)	Completers (*N* = 550)
Women	*n* = 543 (49.8%)	*n* = 275 (50.0%)
Years of education	*M* = 10.7, *SD* = 1.1	*M* = 10.9, *SD* = 1.2
Smoking		
Never	*n* = 501 (45.9%)	*n* = 297 (54.0%)
Yes, but quit	*n* = 465 (42.6%)	*n* = 229 (41.6%)
Currently smoke	*n* = 125 (11.5%)	*n* = 24 (4.4%)
Social (occupational) class		
Professional	*n* = 190 (17.4%)	*n* = 125 (22.7%)
Managerial, technical	*n* = 402 (36.8%)	*n* = 213 (38.7%)
Skilled, nonmanual	*n* = 246 (22.5%)	*n* = 107 (19.5%)
Skilled, manual	*n* = 188 (17.2%)	*n* = 77 (14.0%)
Partly skilled	*n* = 38 (3.5%)	*n* = 16 (2.9%)
Unskilled	*n* = 6 (0.5%)	*n* = 3 (0.5%)

**Table 2.** Longidutinal Summaries by Wave

Characteristic	All comers (*N* = 1,091)				Completers (*N* = 550)			
	70 years	73 years	76 years	79 years	70 years	73 years	76 years^a^	79 years
*N* (% at wave)								
Cardiovascular disease	268 (24.6)	250 (28.9)	236 (33.9)	204 (37.1)	125 (22.7)	155 (28.2)	185 (34.3)	204 (37.1)
Stroke	54 (4.9)	55 (6.4)	73 (10.5)	69 (12.5)	20 (3.6)	33 (6.0)	55 (10.2)	69 (12.5)
Diabetes	91 (8.3)	95 (11.0)	82 (11.8)	71 (12.9)	35 (6.4)	44 (8.0)	59 (10.9)	71 (12.9)
Mean (*SD*)								
Depressive symptoms	2.8 (2.2)	2.6 (2.2)	2.9 (2.3)	3.0 (2.3)	2.7 (2.2)	2.5 (2.2)	2.8 (2.3)	3.0 (2.3)
Matrix Reasoning	13.5 (5.1)	13.2 (5.0)	13.0 (4.9)	12.9 (5.0)	14.4 (5.0)	13.8 (4.9)	13.3 (4.9)	12.9 (5.0)
Block Design	33.8 (10.3)	33.6 (10.1)	32.2 (10.0)	31.2 (9.6)	35.6 (10.2)	34.6 (10.1)	32.7 (9.8)	31.2 (9.6)
Spatial Span	7.4 (1.4)	7.3 (1.4)	7.3 (1.4)	7.1 (1.4)	7.5 (1.4)	7.5 (1.3)	7.4 (1.3)	7.1 (1.4)

Note: *N*s for “all comers” at each wave were 1,091 (70 years), 866 (73 years), 697 (76 years), and 550 (79 years). Completers were individuals who remained in the study through Wave 4.

a Eleven of the completers were absent at Wave 3 (76 years) but returned for Wave 4 (79 years). The corresponding percentages reflect this.

Statistical fit for all models was respectable (see Table 3), with estimates of comparative fit index greater than .95 and root-mean-square error of approximation estimates less than 0.06. BLCSM0, equivalent to a bivariate LGCM, provided unstandardized estimates of static linear change, per 3-year interval, in fluid intelligence (scaled, as described above: *M* = −0.13, 99% confidence interval, or CI = [−0.15, −0.11]) and depressive symptoms (using raw scores: *M* = 0.14, 99% CI = [0.07, 0.21]). Baseline levels of fluid intelligence and depressive symptoms were significantly negatively correlated (*r* = −.17, 99% CI = [−.27, −.07]), whereas static linear changes were not significantly correlated (*r* = −.10, 99% CI = [−.48, .30]). There was also significant between-person variation in levels and linear changes for fluid intelligence and depressive symptoms (see Table A1 in the Appendix). Thus, this first commonly used model predicted that, on average, fluid intelligence decreased by 0.39 (scaled) units and depressive symptoms increased by 0.42 (raw) units across the observed 9-year period and that, whereas baseline levels of depressive symptoms and fluid intelligence were significantly correlated, long-term changes therein were not significantly correlated (despite significant between-person variability in changes).

**Table 3.** Changes in Fit of Bivariate Latent-Change-Score Models With the Addition of Coupling Parameters

Model	Parameters	χ^2^	*df*	CFI	BIC	w(BIC)	RMSEA	Δχ^2^	Δ*df*	*p*
Without covariates										
No coupling	33	186	119	.990	34,194	.251	0.023			
Fluid intelligence → Δdepressive symptoms	34	178	118	.991	34,192	.681	0.022	−8	1	.003
Depressive symptoms → Δfluid intelligence	34	185	118	.990	34,200	.012	0.023	−1	1	.282
Full coupling	35	176	117	.992	34,197	.056	0.021	−10	2	.005

With covariates										
No coupling	77	415	347	.987	21,775	.066	0.019			
Fluid intelligence → Δdepressive symptoms	78	404	346	.989	21,770	.802	0.018	−11	1	.001
Depressive symptoms → Δfluid intelligence	78	411	346	.988	21,777	.024	0.019	−4	1	.040
Full coupling	79	401	345	.989	21,774	.108	0.018	−14	2	.001

Note: Parameter = estimated model parameter; χ^2^ = deviance (−2 × log-likelihood); CFI = comparative fit index; BIC = Bayesian information criterion; w(BIC) = Schwarz weight (i.e., relative probability of model preference); RMSEA = root-mean-square error of approximation; Δχ^2^ = change in model misfit with addition of coupling (or couplings) compared with no-coupling model (lower values = better fit); *p* = *p* value for likelihood-ratio test of change in model fit. The RMSEA 95% confidence interval was within ±.007 for all models.

Comparison tests for the dynamic models, BLCSM1 through BLCSM4, are summarized in Table 3. Models with couplings (BLCSM2–BLCSM4) were tested against the model without couplings (BLCSM1). Likelihood-ratio tests showed significant improvement in fit with addition of the fluid intelligence → Δdepressive symptoms coupling (BLCSM2 vs. BLCSM1). This was true for models tested with and without adjustment for sociodemographic and health covariates (covariate-specific outcomes are reported in Sections S3 and S4 in the Supplemental Material). Addition of the depressive symptoms → Δfluid intelligence coupling did not improve model fit (BLCSM3 vs. BLCSM1). Although the bidirectional couplings model showed better fit than the no-coupling model (BLCSM4 vs. BLCSM1), this appeared to be mostly because of the addition of the fluid intelligence → Δdepressive symptoms coupling alone. We confirmed this in a follow-up test of BLCSM4 versus BLCSM2, which showed a nonsignificant change in model fit (i.e., when adding the depressive symptoms → Δfluid intelligence coupling to BLCSM2). Schwarz weights, w(BICs), favored BLCSM2 above all other models. BLCSM2 accounted for 68.1% (without covariates) and 80.2% (with covariates) of relative predictive accuracy. In contrast, BLCSM3 accounted for only 1.2% (without covariates) and 2.4% (with covariates) of predictive accuracy. Because depressive symptoms variables were positively skewed, we reran the models using log-transformed depressive symptoms scores. Model comparison results from this follow-up analysis (reported in Section S5 in the Supplemental Material) showed a nearly identical outcome. Thus, overall, tests of improvement in model fit clearly favored only inclusion of the fluid intelligence → Δdepressive symptoms coupling (i.e., BLCSM2 was most parsimonious).

**Fig. 2.**
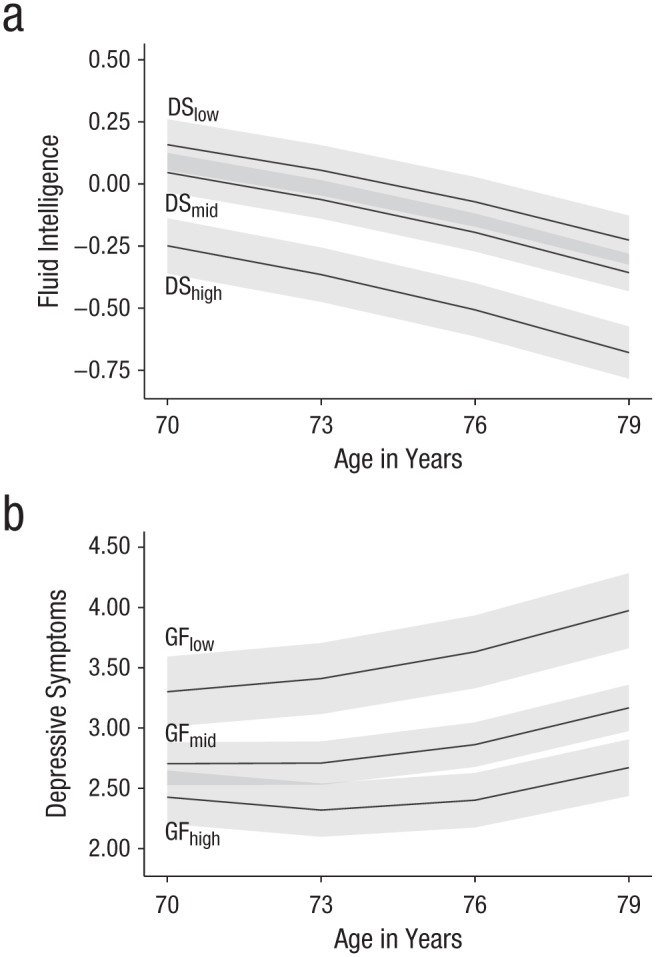
Mean trajectories of fluid-intelligence (GF) performance within subsamples of participants stratified by baseline levels of depressive symptoms (DS; a) and mean trajectories of estimated DS within subsamples stratified by baseline levels of GF (b). Stratification was by interquartile range: low = 0% to 25%, mid = 26% to 75%, high = 76% to 100%. Gray bands indicate 99% confidence intervals.

Estimated trajectories of depressive symptoms and fluid-intelligence scores from BLCSM2 are based on both static parameters (levels, linear changes) and dynamic parameters (auto-proportional and coupling effects). Therefore, to facilitate interpretation, we plot and describe trajectories of fluid-intelligence scores within subsamples of participants stratified by baseline levels of depressive symptoms (see Fig. 2a) and trajectories of depressive symptoms within subsamples of participants stratified by baseline levels of fluid-intelligence scores (see Fig. 2b). On average, fluid-intelligence trajectories for individuals with fewer baseline depressive symptoms were higher but parallel to fluid-intelligence trajectories for individuals with higher baseline levels of depressive symptoms. Thus, between-person differences in fluid intelligence attributed to baseline differences in depressive symptoms were generally preserved across the observed age range (i.e., depressive symptoms did not have a dynamic influence on fluid intelligence). In contrast, depressive-symptoms trajectories for individuals with higher baseline fluid intelligence were lower and increased less than those of individuals with lower baseline fluid intelligence (who had higher and steeper trajectories). This pattern indicates that prior fluid-intelligence scores modified the predicted increases in depressive symptoms, and parameter estimates for BLCSM2 (see Table A1) provide evidence that this was primarily a dynamic (coupling) rather than static (level-slope) effect.

**Fig. 3.**
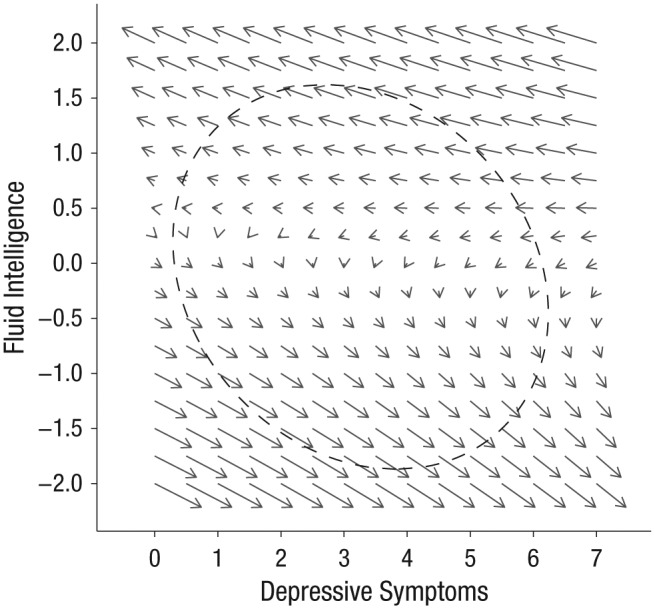
Vector field plot in which arrows show the direction and relative magnitude (longer = stronger effect) of expected changes in fluid intelligence and depressive symptoms. The dashed ellipse demarcates the 90% highest-probability-density region for the estimated true scores.

The effects of static linear changes, auto-proportional effects, and coupling parameters can also be represented in a vector field plot (Boker & McArdle, 1995) to show the expected direction and magnitude of change in depressive symptoms contingent on a given fluid-intelligence score, and vice versa (see Fig. 3). Left-to-right across each row (increasing depressive symptoms), changes in magnitude and in direction (length and orientation of the arrows) along the vertical plane appear relatively small, consistent with the nonsignificant depressive symptoms → Δfluid intelligence coupling. But from top-to-bottom within each column (decreasing fluid-intelligence scores), there is both change in magnitude and flipping of directionality along the horizontal plane, such that higher fluid-intelligence scores predicted subsequent reductions in depressive symptoms, and lower fluid-intelligence scores even more strongly predicted subsequent increases in depressive symptoms. This is consistent with the statistical importance ascribed to the fluid intelligence → Δdepressive symptoms coupling.

To provide effect size estimates for the fluid intelligence → Δdepressive symptoms coupling, we compared the predictions of depressive symptoms scores from the BLCSM without any coupling (BLCSM1) with those from the model with the coupling effect of interest (BLCSM2). Because the coupling effect cannot be estimated prior to the first assessment, the expected scores of depressive symptoms at age 70 years do not differ between BLCSM1 and BLCSM2.^1^ However, the subsequent scores should be expected to differ across these models because of the influence of prior cognitive performance on upcoming changes in depressive symptoms (present in BLCSM2 vs. absent in BLCSM1). The corresponding standardized effect sizes (*d*s) for these differences were 0.02, 99% CI = [−0.08, 0.12] at age 73 years, 0.27, 99% CI = [0.17, 0.37] at 76 years, and 0.88, 99% CI = [0.78, 0.98] at age 79 years. Thus, the estimated coupling association was itself time invariant (fluid intelligence → depressive symptoms γ = −1.37, 99% CI = [−2.40, −0.34]), but when multiplied by cognitive scores that on average worsened with time, it accounted for an accumulated 0.9 standard-deviation increase in depressive symptoms across the observed age range.

## Discussion

We examined time-ordered, reciprocal associations between longitudinal changes in fluid intelligence and depressive symptoms in a large sample (*N* = 1,091) of community-dwelling older adults. Both fluid intelligence and depressive symptoms worsened on average between ages 70 and 79 years. Additionally, there was a significant temporally dependent association, in which poorer fluid intelligence at a given age predicted subsequent increases in depressive symptoms, whereas elevated depressive symptoms were not predictive of subsequent decrements in fluid intelligence. This was shown by improvement in model fit (i.e., with addition of the fluid intelligence → Δdepressive symptoms coupling), by the corresponding parameter estimate for the coupling effect, and by effect-size estimates for differences in depressive-symptoms scores estimated from models with versus without the fluid intelligence → Δdepressive symptoms coupling. Sociodemographic and health-related covariates did not significantly influence the strength of this dynamic association.

Until now, the few longitudinal studies to examine reciprocal lead-lag relations between cognition and depressive symptoms have primarily focused on memory deficits (Aichele & Ghisletta, 2018; Jajodia & Borders, 2011; Perrino et al., 2008). Apart from the difference in cognitive measures used (i.e., fluid intelligence vs. memory), the current results are consistent with these earlier studies in showing that cognitive deficits temporally precede increases in depressive symptoms in older adults. Why this should be so remains an open question. It could be that cognitive deficits and age-related elevation in depressive symptoms share a common basis in neurodegenerative processes or cerebrovascular disease and that cognitive deficits simply manifest behaviorally prior to mood-related changes. There is, however, mixed evidence as to whether cognition-depression associations persist after statistical control for vascular diseases and related risk factors (e.g., Jajodia & Borders, 2011; van den Kommer et al., 2013). The current results show that statistical adjustment for prior diagnosis of diabetes, cardiovascular disease, and stroke had little effect on estimates of temporal associations between fluid intelligence and depressive symptoms. Notwithstanding, prior studies using subsamples of LBC1936 participants have demonstrated that reduction of cerebral white-matter structural connectivity is associated with both elevated depressive symptoms (McIntosh et al., 2013) and decline in general fluid intelligence (Ritchie et al., 2015).

Genetic susceptibility may also play a role in depression-cognition associations. Kievit et al. (2016) proposed that many small, independent genetic influences act through numerous mediating endophenotypes (e.g., such as cerebral white-matter loss) to affect “downstream” behavioral phenotypes, such as fluid intelligence. Thus, the contribution of a single genetic marker within the causal cascade will be largely obscured when joined by influences more proximal to a given behavioral phenotype. This implies that identification of genetic risk factors that affect the associations between fluid intelligence and depression will require sophisticated research methodologies and very large sample sizes. The scale of such a study was demonstrated in recent work from Hill and colleagues (2018), who used a meta-analytic approach to pool information from two large genome-wide association studies (for an effective *N* = 248,482) to identify 187 loci (538 genes) that accounted for approximately 7% of variation in intelligence. This methodology and result are evidence that the sample size and analytical framework necessary to identify genes influencing cognition–depression associations are well outside the scope of the current study.

More pragmatically, preserved fluid intelligence is strongly linked to independent functioning in activities of daily life (Tucker-Drob, 2011). Older adults may become distressed with increased awareness of their own cognitive decline and the implications for day-to-day functioning (Vinkers et al., 2004). There is currently little evidence to support this hypothesis (Carpenter et al., 2008), but, if true, treatment for depression could protect against diminished quality of life for individuals already confronted with cognitive impairment (Perrino et al., 2008). Alternatively, fluid-intelligence-related impairment of behaviors important for self-care and day-to-day functioning may give rise to undesirable conditions (social isolation, financial instability, poor health) that in turn lead to worsening emotional well-being. To disambiguate these hypotheses, it will be important for cognition-depression studies to include measures of subjective mental decline and also for such studies to take advantage of statistical models that allow testing time-ordered relations alongside mediating or moderating influences (e.g., difficulties in activities of daily living).

Along these lines, inclusion of measures of subjective cognitive decline may have allowed for a stronger interpretation of the fluid intelligence → Δdepressive symptoms coupling. Note, however, that in the BLCSM, couplings are fixed effects (estimated only at the group level) and thus cannot be used directly in predictor–outcome associations. This means that a multiple-groups framework is needed to compare cognition–depression couplings across subsamples defined by a categorical moderating variable, such as high versus low subjective cognitive decline. Similarly, it would be worthwhile to estimate time-ordered effects of mediating variables, such as difficulties in activities of daily living. This would require modeling covariates as processes in their own right. Both approaches would add substantial statistical complexity to an already complex series of analyses. We therefore view further explorations of moderating and mediating variables in the associations between fluid intelligence and depression as topics for future study.

Other limitations of the study include use of a single self-report measure of depressive symptoms, a rather homogeneous participant sample with respect to socioeconomic status and ethnicity (all were Caucasian), and the observation of change associations across a relatively narrow age range (approximately 70–79 years). Interpretation of the findings is further constrained in that depressive symptoms were assessed at relatively long (3-year) intervals. Further discussion of strengths and limitations of the Lothian Birth Cohort Studies can be found in Deary et al. (2012).

## Conclusion

To our knowledge, this is the first study to examine time-ordered, reciprocal associations between changes in depressive symptoms and fluid intelligence. The primary contribution of the study is that it demonstrates that fluid-intelligence decrements beginning at age 70 years precede increases in depressive symptoms, whereas elevated depressive symptoms in later life do not appear to affect subsequent changes in fluid intelligence. Statistical adjustment for sociodemographic and health-related covariates had only a small effect on the strength of this association. We conclude that proactive monitoring for cognitive decrements in later life may expedite interventions to reduce associated increases in depression risk.

## Supplemental Material

AicheleSupplementalMaterial – Supplemental material for Fluid Intelligence Predicts Change in Depressive Symptoms in Later Life: The Lothian Birth Cohort 1936 Click here for additional data file. Supplemental material, AicheleSupplementalMaterial for Fluid Intelligence Predicts Change in Depressive Symptoms in Later Life: The Lothian Birth Cohort 1936 by Stephen Aichele, Paolo Ghisletta, Janie Corley, Alison Pattie, Adele M. Taylor, John M. Starr and Ian J. Deary in Psychological Science

## Appendix

**Table A1.** Parameter Estimates From Two Bivariate Latent-Change-Score Models: BLCSM0 and BLCSM2

Parameter type and parameter	BLCSM0 (bivariate latent change without couplings or auto-proportional effects)		BLCSM2 (bivariate latent change with fluid intelligence → depressive symptoms coupling)	
	Estimate	99% CI	Estimate	99% CI
Means				
Level of fluid intelligence	0.008	[−0.067, 0.083]	0.000	[−0.075, 0.075]
Slope of fluid intelligence	−0.131	[−0.152, −0.110]	−0.109	[−0.148, −0.070]
Level of depressive symptoms	2.730	[2.560, 2.900]	2.784	[2.609, 2.959]
Slope of depressive symptoms	0.139	[0.069, 0.209]	0.540	[−0.861, 1.941]
Variances				
Level of fluid intelligence	0.632	[0.521, 0.743]	0.628	[0.517, 0.739]
Slope of fluid intelligence	0.008	[0.003, 0.013]	0.040	[−0.042, 0.122]
Level of depressive symptoms	3.436	[2.900, 3.972]	3.452	[2.906, 3.998]
Slope of depressive symptoms	0.138	[0.056, 0.220]	1.151	[−0.523, 2.825]
Covariances (standardized)				
Level of fluid intelligence with slope of fluid intelligence	−0.235	[−0.427, −0.023]	−0.941	[−0.957, −0.919]
Level of depressive symptoms with slope of depressive symptoms	−0.077	[−0.291, 0.144]	−0.121	[−0.732, 0.598]
Level of fluid intelligence with level of depressive symptoms	−0.173	[−0.269, −0.074]	−0.164	[−0.262, −0.062]
Level of fluid intelligence with slope of depressive symptoms	−0.145	[−0.341, 0.063]	0.870	[0.781, 0.924]
Level of depressive symptoms with slope of fluid intelligence	−0.076	[−0.306, 0.163]	0.111	[−0.054, 0.270]
Slope of fluid intelligence with slope of depressive symptoms	−0.103	[−0.475, 0.300]	−0.822	[−0.897, −0.702]
Proportional changes (β)				
Fluid intelligence → Δfluid intelligence			0.219	[−0.072, 0.510]
Depressive symptoms → Δdepressive symptoms			−0.193	[−0.703, 0.317]
Coupling effects (γ)				
Fluid intelligence → Δdepressive symptoms			−1.369	[−2.397, −0.341]
Fluid intelligence factor loadings (Wave 1, standardized)				
Block Design	0.796	[0.752, 0.840]	0.796	[0.752, 0.840]
Matrix Reasoning	0.706	[0.671, 0.741]	0.703	[0.657, 0.749]
Spatial Span (forward and backward)	0.551	[0.499, 0.603]	0.551	[0.499, 0.603]
Residual variances				
Block Design	0.365	[0.301, 0.429]	0.364	[0.300, 0.428]
Matrix Reasoning	0.490	[0.433, 0.547]	0.492	[0.435, 0.549]
Spatial Span (forward and backward)	0.687	[0.628, 0.746]	0.686	[0.627, 0.745]
Depressive symptoms	1.606	[1.446, 1.766]	1.583	[1.423, 1.743]

Note: Values in brackets are 99% confidence intervals. Slope refers to static linear change per 3-year interval (between ages 70 and 79 years). Except where noted, estimates shown are unstandardized and based on scaled scores for fluid intelligence (see the Method section) and raw scores for depressive symptoms. Proportional changes and coupling effects reflect scores at time *t* as predictive of change in score between time *t* and *t* + 3 years.

## Notes

1. In this follow-up analysis, parameters for BLCSM1 were fixed to the corresponding estimates obtained from BLCSM2 so that estimated scores at the first measurement occasion were equal across models.
