# Supplementary material for: Fluid Intelligence Predicts Change in Depressive Symptoms in Later Life: The Lothian Birth Cohort 1936
Source: Psychol Sci. 2018 Oct 25;29(12):1984–95. doi: 10.1177/0956797618804501 (PMC6291904; doi:10.1177/0956797618804501)
Supplement: AicheleSupplementalMaterial – Supplemental material for Fluid Intelligence Predicts Change in Depressive Symptoms in Later Life: The Lothian Birth Cohort 1936 [file AicheleSupplementalMaterial.pdf]

Online Supplemental Material

S1. Correlations among cognitive items and depressive symptoms based on raw data

	BD1	BD2	BD3	BD4	MR1	MR2	MR3	MR4	SS1	SS2	SS3	SS4	DS1	DS2	DS3	DS4
**BD1**	1.00															
**BD2**	0.77	1.00														
**BD3**	0.76	0.76	1.00													
**BD4**	0.72	0.76	0.79	1.00												
**MR1**	0.57	0.53	0.57	0.53	1.00											
**MR2**	0.56	0.54	0.55	0.52	0.65	1.00										
**MR3**	0.56	0.53	0.58	0.51	0.64	0.65	1.00									
**MR4**	0.55	0.55	0.58	0.58	0.64	0.66	0.65	1.00								
**SS1**	0.40	0.41	0.43	0.36	0.38	0.36	0.40	0.41	1.00							
**SS2**	0.42	0.45	0.45	0.42	0.39	0.37	0.40	0.41	0.56	1.00						
**SS3**	0.37	0.39	0.45	0.35	0.33	0.37	0.43	0.43	0.59	0.57	1.00					
**SS4**	0.41	0.41	0.45	0.44	0.38	0.37	0.42	0.44	0.58	0.59	0.60	1.00				
**DS1**	-0.10	-0.11	-0.13	-0.14	-0.10	-0.07	-0.14	-0.08	-0.12	-0.10	-0.09	-0.16	1.00			
**DS2**	-0.12	-0.14	-0.12	-0.14	-0.07	-0.06	-0.11	-0.09	-0.13	-0.11	-0.11	-0.12	0.67	1.00		
**DS3**	-0.18	-0.19	-0.22	-0.22	-0.14	-0.11	-0.20	-0.17	-0.19	-0.18	-0.18	-0.20	0.63	0.70	1.00	
**DS4**	-0.15	-0.15	-0.18	-0.17	-0.09	-0.09	-0.16	-0.11	-0.15	-0.13	-0.14	-0.14	0.61	0.62	0.73	1.00

Note: BD = block design. MR = matrix reasoning. SS = spatial span. DS = depressive symptoms. Numbers that are part of each acronym correspond to measurement waves (e.g., BD1 = block design at wave 1).

S2. Longitudinal structural factor analysis: Factorial invariance of fluid intelligence

Model Invariance	Params	CFI	BIC	RMSEA [95%CI]	SRMR	χ^2	$\Delta\chi^2$	Δdf	*p*
Strict	23	0.94	21589	0.07 [0.06, 0.08]	0.10	21429			
Strong/Scalar	32	0.94	21638	0.08 [0.07, 0.08]	0.10	21415	-14	9	0.13
Weak/Metric	41	0.99	21409	0.04 [0.03, 0.05]	0.10	21122	-293	9	0.00
Configural	47	0.99	21446	0.04 [0.03, 0.05]	0.10	21118	-4	6	0.61

Note: Params = estimated model parameters. χ^2 = deviance (-2 * log-likelihood). CFI = comparative fit index. BIC = Bayesian information criterion. RMSEA = root mean square error of approximation. $\Delta\chi^2$ = change in model misfit with addition of coupling(s) compared to the immediately preceding model (from top to bottom in order of testing; decreases indicate improved fit). *p* = p-value for likelihood ratio test of change in model fit.

Analyses used data from 1088 observations. The base model (strict invariance) corresponds to the fluid intelligence measurement structure (i.e., Gf factors and items/loadings) of the model shown in Figure 1 of the main manuscript. Here, Gf factor scores were allowed to covary rather than being regressed onto latent change parameters (level, slope, change scores). Degree of factorial invariance across measurement occasions was sequentially tested, starting with the most rigorously constrained model. Under strict invariance, constraints were placed on item-factor loadings, item means, and item unique variances. Under strong/scalar invariance, constraints were placed on item-factor loadings and item means. Under weak/metric invariance, constraints were placed on item-factor loadings. Under configural invariance, the above parameters were freely estimated.

Absolute model fit criteria (CFI, RMSEA, SRMR) indicated that the item-factor representation and scaling were consistent (i.e., were strictly factorial invariant) across measurement occasions (CFI ≥ .90; RMSEA ≤ .10; SRMR ≤ .10). Tests of change in model fit showed that freeing unique variances (strong vs. strict invariance) did not significantly improve model fit, that freeing item means (weak/metric vs. strong invariance) did significantly improve model fit, and that freeing factor loadings (configural vs. weak invariance) did not further improve model fit. These tests of relative fit would favor weak/metric invariance if absolute fit criteria were not acceptable under stricter invariance (they were). Note that meeting weak/metric invariance is a sufficient requirement for the estimation of the BLCSM; Nevertheless, given that it held statistically, we estimated the BLCSM under strict invariance. Further information on tests of factorial invariance can be found in Widaman and Reise (1997), and information on comparative fit statistics can be found in Hu and Bentler (1999).

Hu, L., & Bentler, P. M. (1999). Cutoff criteria for fit indexes in covariance structure analysis: conventional criteria versus new alternatives. *Structural Equation Modeling: A Multidisciplinary Journal*, *6*, 1–55. doi:10.1080/10705519909540118

Widaman, K. F., & Reise, S. P. (1997). Exploring the measurement invariance of psychological instruments: Applications in the substance use domain. In K. J. Bryant, M. Windle, & S. G. West (Eds.), *The science of prevention: Methodological advances from alcohol and substance abuse research* (pp. 281–324). Washington, DC: American Psychological Association.

S3. Time-invariant predictors of levels and slopes of fluid intelligence and depressive symptoms

Predictors	Estimate	S.E.	*p*
			Outcome: Level of Fluid Intelligence
Sex = Female	-0.297	0.060	0.000
Years of Education	0.174	0.030	0.000
Social Class[a]	-0.131	0.037	0.000
Smoker (former)	-0.044	0.061	0.467
Smoker (current)	-0.198	0.144	0.169
			Outcome: Slope of Fluid Intelligence
Sex = Female	0.122	0.055	0.026
Years of Education	-0.072	0.030	0.018
Social Class[a]	0.044	0.028	0.124
Smoker (former)	0.009	0.029	0.757
Smoker (current)	0.058	0.073	0.428
			Outcome: Level of Depressive Symptoms
Sex = Female	-0.319	0.189	0.091
Years of Education	-0.043	0.091	0.640
Social Class[a]	0.036	0.115	0.753
Smoker (former)	0.101	0.193	0.601
Smoker (current)	0.073	0.459	0.874
			Outcome: Slope of Depressive Symptoms
Sex = Female	-0.587	0.238	0.014
Years of Education	0.348	0.127	0.006
Social Class[a]	-0.303	0.123	0.014
Smoker (former)	-0.084	0.143	0.557
Smoker (current)	-0.215	0.359	0.549

Note: Estimates are shown unstandardized.

[a] Social Class was based on the Office of Population Censuses and Surveys' Classification of Occupations (1980), which consists of 6 ordered groupings, professional to unskilled labor, such that lower numbered groups indicate higher social class. Social Class was treated as a continuous variable in the model.

Office-of-Population-Censuses-and-Surveys (1980). *Classification of occupations 1980*. London: Her Majesty's Stationery Office.

S4. Time-varying predictors of fluid intelligence scores and depressive symptoms

Predictors	Estimate (SE) by Outcome: Fluid Intelligence	Depressive Symptoms
	Wave 1	
Cardiovascular Disease	0.011 (0.028)	0.262 (0.239)
Stroke	0.088 (0.064)	0.349 (0.569)
Diabetes	-0.044 (0.056)	0.520 (0.406)
	Wave 2	
Cardiovascular Disease	-0.030 (0.029)	0.247 (0.162)
Stroke	-0.044 (0.058)	0.175 (0.329)
Diabetes	0.028 (0.054)	0.053 (0.271)
	Wave 3	
Cardiovascular Disease	-0.041 (0.030)	0.126 (0.140)
Stroke	-0.076 (0.051)	0.337 (0.228)
Diabetes	-0.018 (0.055)	0.334 (0.240)
	Wave 4	
Cardiovascular Disease	0.034 (0.043)	-0.071 (0.168)
Stroke	0.057 (0.062)	-0.199 (0.235)
Diabetes	-0.028 (0.062)	-0.201 (0.244)

Note: Estimates are shown unstandardized. *p*-values corresponding to these estimates were in all cases lager than 0.15

S5. Changes in BLCSM fit with addition of coupling parameters (using log-transformed depressive symptoms scores)

Model	Params	$\chi 2$	df	CFI	BIC	RMSEA	$\Delta\chi 2$	Δdf	*p*
no coupling	33	200	119	.988	28311	.025			
Gf $\rightarrow$ ΔDS_t	34	192	118	.989	28311	.024	-8	1	.005
$DS_t \rightarrow \Delta Gf$	34	197	118	.988	28316	.025	-3	1	.089
full coupling	35	190	117	.989	28315	.024	-10	2	.005

Note: Gf = fluid intelligence. DS_t = depressive symptoms, log-transformed. Arrows designate direction of coupling effect. Params = estimated model parameters. χ^2 = deviance (-2 * log-likelihood). CFI = comparative fit index. BIC = Bayesian information criterion. RMSEA = root mean square error of approximation. $\Delta\chi^2$ = change in model misfit with addition of coupling(s) compared to the no-coupling model (lower values correspond to better fit). *p* = p-value for likelihood ratio test of difference in model fit. RMSEA 95%CI was within ±.007 for all models.
